# Genome Sequences of Five B1 Subcluster Mycobacteriophages

**DOI:** 10.1128/genomeA.00968-13

**Published:** 2013-11-27

**Authors:** Donald P. Breakwell, E. Zane Barrus, Alex B. Benedict, Alicia K. Brighton, Joshua N. B. Fisher, Adam V. Gardner, Brittany J. Kartchner, Kara C. Ladle, Bryce L. Lunt, Bryan D. Merrill, John D. Morrell, Sandra H. Burnett, Julianne H. Grose

**Affiliations:** Department of Microbiology and Molecular Biology, Brigham Young University, Provo, Utah, USA

## Abstract

Mycobacteriophages infect members of the *Mycobacterium* genus in the phylum *Actinobacteria* and exhibit remarkable diversity. Genome analysis groups the thousands of known mycobacteriophages into clusters, of which the B1 subcluster is currently the third most populous. We report the complete genome sequences of five additional members of the B1 subcluster.

## GENOME ANNOUNCEMENT

More than 3,600 mycobacteriophages have been isolated to date, and >298 of their genome sequences are accessible in GenBank (see http://www.phagesdb.org). These numbers reflect the significant biological diversity among bacteriophages, especially because all of these phages infect a single host, *Mycobacterium smegmatis* strain mc^2^155 (1). Dot-plot analyses, average nucleotide identities, gene content analyses, and pairwise genome analyses of mycobacteriophage genomes grouped the phages into 17 clusters and 39 subclusters (2). Of these, the B1 subcluster of mycobacteriophages is one of the most populous, accounting for approximately 10% of the mycobacteriophage genome sequences found in GenBank. We report here the genome sequences of five novel B1 subcluster phages.

The phages were isolated and characterized by students in the Freshman Research Initiative course at Brigham Young University (BYU). The program is part of the Howard Hughes Medical Institute Science Education Alliance’s Phage Hunters Advancing Genomics and Evolutionary Sciences Program (SEA-PHAGES). In the course, each student isolated and plaque purified a single mycobacteriophage using *M. smegmatis* mc^2^155 as the host. After three rounds of plaque purification, followed by amplification to a high titer, the genomic DNA was isolated (Wizard DNA Clean-Up System, Promega Corp., Madison, WI) and submitted for 454 pyrosequencing (Roche) at the BYU DNA Sequencing Center. Students in the program named the phages and annotated the genome sequences. The teaching assistants and faculty checked the annotated genome sequences prior to their submission to GenBank.

Each genome had at least 65-fold coverage (average, 119.8-fold). Assembly was conducted using GS *de novo* assembler 2.6 (Newbler; Roche) and verified with Consed version 19 (3). Base one was called by alignment with the other B1 phages. The genome sequences were annotated using GeneMark coding potential maps with *M. tuberculosis* CDC 1551 as a reference strain (4) and DNA Master software (J. G. Lawrence lab [http://cobamide2.bio.pitt.edu]), which integrates Glimmer 3.02, GeneMark, BLAST, Shine-Dalgarno (SD) position-weighted scores, and general annotation tools.

The sizes (average, 68,800 bp) and organization of the genomes (approximately 100 open reading frames) are consistent with those of other B1 subcluster mycobacteriophages catalogued in GenBank to date. As with other B1 subcluster phages, all five phages reported here are *Siphoviridae* with double-stranded DNA (dsDNA) circularly permuted genomes. The analysis of an increasing data set of phage genomes provides insights not only into the diversity of bacteriophages and phage-host interactions but also into their evolution. Additional information on these phage and their genomes is available at http://www.phagesdb.org. Subsequent reports will describe the specific attributes and comparative analyses of the phages announced herein.

### Nucleotide sequence accession numbers.

The GenBank accession numbers and pertinent traits of the five genome sequences are reported in Table 1.

**TABLE 1:** Data for five new B1 subcluster mycobacteriophage genome sequences

Phage	GenBank accession no.	Length (bp)	% G+C	No. of ORFs	Year isolated
Alex	JX649100	68,910	66.52	105	2009
Gyarad	JX649099	68,004	66.49	100	2009
Nacho	JX649098	69,321	66.56	104	2011
Piglet	JX649097	68,992	66.54	101	2011
Serpentine	JX649096	68,884	66.51	104	2010
